# Development of Bexarotene Analogs for Treating Cutaneous T-Cell Lymphomas

**DOI:** 10.3390/cells12212575

**Published:** 2023-11-04

**Authors:** Ankedo Warda, Lech J. P. Staniszewski, Zhela Sabir, Sarah Livingston, Michael Sausedo, Sabeeha Reshi, Eyal Ron, Michael T. Applegate, Dena Haddad, Madleen Khamisi, Pamela A. Marshall, Carl E. Wagner, Peter W. Jurutka

**Affiliations:** 1 School of Mathematical and Natural Sciences, Arizona State University, Glendale, AZ 85306, USA; anwarda@arizona.edu (A.W.); lech@arizona.edu (L.J.P.S.); zhelasa@gmail.com (Z.S.); sarahklivingston5@gmail.com (S.L.); msausedo@asu.edu (M.S.); sreshi@asu.edu (S.R.); mtapplegate@arizona.edu (M.T.A.); denahaddad227@gmail.com (D.H.); madleenkhamisi@gmail.com (M.K.); pamela.marshall@asu.edu (P.A.M.); carl.wagner@asu.edu (C.E.W.); 2 College of Medicine, University of Arizona, Phoenix, AZ 85004, USA; ron.eyal@mayo.edu; 3 College of Medicine, University of Arizona, Tucson, AZ 85724, USA

**Keywords:** rexinoids, RXR, cutaneous T-cell lymphoma, SAR, cancer

## Abstract

Bexarotene, a drug approved for treatment of cutaneous T-cell lymphoma (CTCL), is classified as a rexinoid by its ability to act as a retinoid X receptor (RXR) agonist with high specificity. Rexinoids are capable of inducing RXR homodimerization leading to the induction of apoptosis and inhibition of proliferation in human cancers. Numerous studies have shown that bexarotene is effective in reducing viability and proliferation in CTCL cell lines. However, many treated patients present with cutaneous toxicity, hypothyroidism, and hyperlipidemia due to crossover activity with retinoic acid receptor (RAR), thyroid hormone receptor (TR), and liver X receptor (LXR) signaling, respectively. In this study, 10 novel analogs and three standard compounds were evaluated side-by-side with bexarotene for their ability to drive RXR homodimerization and subsequent binding to the RXR response element (RXRE). In addition, these analogs were assessed for proliferation inhibition of CTCL cells, cytotoxicity, and mutagenicity. Furthermore, the most effective analogs were analyzed via qPCR to determine efficacy in modulating expression of two critical tumor suppressor genes, ATF3 and EGR3. Our results suggest that these new compounds may possess similar or enhanced therapeutic potential since they display enhanced RXR activation with equivalent or greater reduction in CTCL cell proliferation, as well as the ability to induce ATF3 and EGR3. This work broadens our understanding of RXR–ligand relationships and permits development of possibly more efficacious pharmaceutical drugs. Modifications of RXR agonists can yield agents with enhanced biological selectivity and potency when compared to the parent compound, potentially leading to improved patient outcomes.

## 1. Introduction

There are varieties of T- and B-cell neoplasms that may affect the skin, either primarily or secondarily. Primary cutaneous lymphomas are a heterogenous group of extranodal non-Hodgkin lymphomas that include both cutaneous T-cell lymphomas (CTCLs) and cutaneous B-cell lymphomas (CBCLs) [1]. Upon initial diagnosis, these neoplasms are present strictly in the integumentary system without evidence of extracutaneous involvement. The classification of primary cutaneous lymphomas is crucial due to the similar histological properties shared with systemic lymphomas that may secondarily involve the skin. Primary and secondary cutaneous lymphomas demonstrate completely different clinical behaviors and prognoses, and therefore require different treatment plans. Approximately 75% of primary cutaneous lymphomas are derived from T-cells, of which two-thirds are further characterized as either Mycosis fungoides (MF) or Sézary syndrome (SS) [1,2,3].

Classic MF tends to be a more indolent disease which slowly progresses over the course of several years to decades, advancing through stages including patches, plaques, and tumors [4,5]. Patients with MF present with well-defined and pruritic erythematous patches and plaques often distributed in sun-protected areas. These lesions have varying sizes and levels of desquamation, can ulcerate in advanced disease, and very rarely present as hypopigmented lesions in younger individuals and those with high skin melanin [6]. Some patients may present with concurrent patches, plaques, and tumors and although tumors often signify later-stage disease, they may also occur de novo [7].

Although less common than MF, SS is another distinct and more aggressive type of CTCL. Classic SS is characterized by intensely pruritic and generalized skin involvement with erythroderma, lymphadenopathy, and leukemic spread of malignant CD4+ T cells [6,7]. SS often presents de novo, however, and may also develop from long-standing MF, known as “SS preceded by MF” or “secondary SS” [7]. Erythrodermic lesions in SS may range from mild erythema to generalized exfoliative dermatitis and fissuring involving the palms and soles, and are often misdiagnosed as allergy, atopic dermatitis, adverse drug reactions, or chronic contact dermatitis [8].

While its pathophysiology is quite complex, CTCL is the result of malignant transformation of skin-homing/resident T-cells [1] and may be more simply explained in terms of its “microenvironment” and “macroenvironment”. The observation that T-cell costimulatory signals support the growth of malignant T-cells in vitro highlights the importance of extrinsic factors present in the tumor microenvironment [9,10]. Furthermore, gene expression profiling and immunohistochemistry-based studies have demonstrated the important role of nonmalignant cells, including lymphoma-associated macrophages and dendritic cells, which may be recruited into the tumor microenvironment by tumor-derived chemokines [11,12]. These nonmalignant cells are then able to promote tumorigenesis via direct mechanisms like the production of factors involved in tumor cell growth and survival, and indirect mechanisms such as the promotion of angiogenesis and suppression of host antitumor immunity [13]. Contributing to the significant morbidity and mortality associated with the infectious complications often seen in CTCL is widespread impairment of cellular immunity—the tumor “macroenvironment” [14]. Roughly 50% of CTCL patients, especially those with advanced disease, eventually succumb to infection [15,16,17]. This phenomenon is due to both quantitative and qualitative defects in immune system functions, including natural killer cells [18,19], dendritic cells [20], and T-cell mediated immunity [21,22,23] amongst other components such as the loss of T-cell repertoire, analogous to that observed in HIV infection, and loss of T-cell receptor diversity [14,24].

According to the World Health Organization-European Organization for Research and Treatment of Cancer classification system, disease staging dictates the choice of treatment in both MF and SS [1]. Treatment options range from skin-directed therapies (SDTs) to biologic systemic therapies and, of course, chemotherapy. In the early stages of the disease, patients often respond well to SDTs alone. In more advanced CTCL, however, SDTs may be used in combination with systemic therapy. Common SDTs include alkylating agents, phototherapy, photodynamic therapy, and electron beam therapy [25]. The selection of systemic therapies for CTCL is broad, and many agents may be used in combination, including cytokines such as IFNα and IFNγ, methotrexate, denileukin diftitox, histone deacetylase inhibitors, monoclonal antibodies, forodesine, stem cell transplants, and bexarotene.

Bexarotene is classified as a rexinoid (a subclass of retinoids) with binding specificity for the retinoid X receptor (RXR) and was FDA approved in 1999 for the treatment of advanced CTCL refractory to at least one systemic treatment. Both retinoids and rexinoids are immunomodulating agents that are structurally similar to vitamin A. The RXR isoforms (α, β, γ) are differentially expressed in every type of human tissue where they function as transcriptional regulators, oftentimes in partnership with other nuclear receptors (NRs). Bexarotene works predominantly by binding to RXR, inducing RXR homodimerization, and subsequently translocating the ligand bound homodimer complex onto its respective RXR response element (RXRE), found upstream of genes involved in a broad range of cellular processes. When bound to RXRE, the RXR homodimer is able to act as a transcriptional regulator, modulating the expression of genes responsible for cell differentiation and proliferation, apoptosis, and insulin sensitization [26]. Via these mechanisms, bexarotene has been found to be quite effective in inducing apoptosis and inhibiting proliferation in CTCL cell lines and peripheral blood T-cells from patients with SS [27,28]. Additionally, bexarotene blunts malignant T-cell trafficking to the skin through downregulation of chemokine receptor type 4 and E-selectin expression [29] further contributing to its efficacy in treating CTCL.

**Figure 1:**
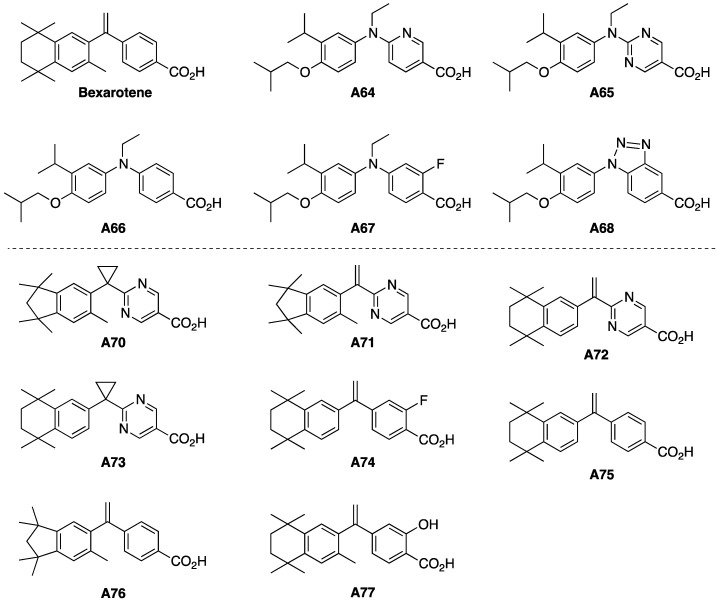
Bexarotene, Generation 6, and Generation 7 Compounds.

Despite its success in treating refractory CTCL, bexarotene is not without its limitations. For example, in two clinical trials untoward effects occurred including hypertriglyceridemia, hypercholesterolemia, and hypothyroidism [30]. Even topical bexarotene can cause significant dermal irritation leading to patient withdrawal from the trial [31]. These pleiotropic effects arise due to the unique and necessary role RXR plays in the function of other NRs. A large body of the literature has elucidated two primary heterodimer classifications in which RXR associates with and potentiates other NRs—known as permissive and non-permissive RXR heterodimers [32]. In the case of permissive RXR heterodimers, either the RXR agonist or the heteropartner’s agonist can activate the heterodimer complex. Conversely, non-permissive RXR heterodimers require only the heteropartner’s agonist to activate the heterodimer complex. The retinoic acid receptor (RAR) and thyroid hormone receptor (TR) RXR heterodimers have been characterized as non-permissive whereas the liver X receptor (LXR) RXR heterodimer is known to be permissive. Therefore, when patients are treated with bexarotene, a potent rexinoid, hyperlipidemia and hypertriglyceridemia may occur as the RXR-LXR heterodimer can become activated in the presence of the rexinoid. On the other hand, cutaneous toxicity and hypothyroidism are seen in patients given bexarotene due to titration of a finite pool of RXR, preventing proper formation and function of RXR-RAR and RXR-TR—a process known as cross-receptor squelching [33]. Thus, enhanced selectivity and potency have become the two overarching themes regarding the development of rexinoid therapeutics with less receptor crosstalk and milder pleiotropic profiles [34]. In this study, ten rexinoid analogs of bexarotene and three standards from the literature (Figure 1), whose complete chemical synthesis and initial characterization we recently reported [35], are evaluated for their biological selectivity and potency as alternative candidates to bexarotene for the treatment of CTCL.

Due to structural/chemical differences in our novel panel of analogs (Figure 1), the compounds were separated into “functional” generation 6 (**A64**–**A68**) and generation 7 (**A70**–**A77**) groupings, and we maintain this separate distinction for these compounds throughout our results and discussion below. Generation 6 includes the model compound standard from the literature **A64** (NEt-4IB) [36] as well as **A65**–**A68**, which are all structurally related to the model compound. Generation 7 includes **A70**–**A77** which were modeled more closely after bexarotene and reported model compounds **A75** [37] and **A76** [38]. All synthetic experimentals as well as characterization data (H-NMR/13C-NMR spectra, high resolution mass spectrometry, and HPLC traces) for the generation 6 (**A64**–**A68**) and generation 7 (**A70**–**A77**) compounds have been reported [35] and are freely available. The analogs, **A65**–**A68**, of the NEt-4IB (**A64**) standard compound were of interest to examine for their potential to activate RXR and impact CTCL proliferation since Kakuta’s group described the standard NEt-4IB (**A64**) compound in generation 6 as a partial RXR agonist [36]. Indeed, a partial RXR agonist in the generation 6 series that could avoid triggering severe side effects but inhibit CTCL proliferation could represent a potentially better drug compound than bexarotene. The generation 7 compounds were modeled more closely after the parent bexarotene model compound, with modified features such as aliphatic ring contractions, cyclopropyl ring-linking substitutions for bexarotene’s vinyl ring-linking group, demethylations, heterocycle aromatic substitutions, and halogenation/hydroxylation of the carboxylic acid bearing aromatic ring. Compounds **A70**–**A73** are analogs of novel pyrimidine-bexarotene and pyrimidine-LG100265 analogs that we reported previously, but they possess additional variations such as aliphatic ring contractions in the case of **A70** and **A71** or demethylations in the case of **A72** and **A73**. We hypothesized that compounds in generation 7 would exhibit a range of RXR activation more similar to the model bexarotene, **A75**, and **A76** compounds with similar or superior abilities to inhibit CTCL proliferation.

## 2. Experimental

### 2.1. Mammalian Two-Hybrid Assay

Human embryonic kidney (HEK-293) cells were obtained from the American Type Culture Collection (ATCC, Manassas, VA, USA, Catalog #CRL-1573). The cells were plated at 80,000 cells per well and allowed to incubate for 24 h in a 24-well plate, maintained in Dulbecco’s modified Eagle’s medium (DMEM) + glutamine + sodium pyruvate (Mediatech, Manassas, VA, USA) containing 10% fetal bovine serum (FBS) (Atlanta Biologicals, Atlanta, GA, USA), supplemented with 100 μg/mL streptomycin and 100 unit/mL penicillin (Gibco, Gaithersberg, MD, USA) at 37 °C and 5% CO_2_. Cells were then cotransfected using a human RXR-binding domain (BD) vector (bait), a human RXR-activation domain (AD) vector (prey), pFR-Luc, and renilla control plasmid. Transfection was performed using 1.25 μL of Polyjet (Signagen, Gaithersberg, MD, USA) per well and allowed to incubate for an additional 24 h. The cells were then treated with ethanol, bexarotene, or the indicated analog at a final concentration of 100 nM and incubated for 24 h. Subsequently, rexinoid activity was measured by luciferase output utilizing a dual-luciferase reporter assay system according to the manufacturer’s instructions (Promega, Madison, WI, USA) via a Sirus luminometer (Berthold Detection Systems, Pforzheim, Germany). At least three independent assays were conducted with triplicate samples for each treatment group. The value for the positive control, bexarotene, was set to 100%.

### 2.2. RXRE-Mediated Transcription Assay

HEK-293 cells were plated at 80,000 cells per well and allowed to incubate for 24 h in a 24-well plate while maintained as described above. Cells were then cotransfected using 250 ng of RXRE luciferase reporter gene (RXRE from the naturally occurring responsive element in rat cellular retinol binding protein II gene: 5′-AAAATGAACTGTGACCTGTGACCTGTGACCTGTGAC-3′, in which DR1-responsive elements are underlined), 25 ng of pSG5- human RXRα, and 20 ng of renilla control plasmid using 1.25 μL of Polyjet per well and allowed to incubate for an additional 24 h. The cells were then treated with ethanol, bexarotene, or an analog at a final concentration of 100 nM and incubated for 24 h. Subsequently, the rexinoid activity was measured by luciferase output as described above. At least three independent assays were conducted with triplicate samples for each treatment group. The value for the positive control, bexarotene, was set to 100%.

### 2.3. Proliferation Assay

Human T-cell lymphoma (Hut78) cells were obtained from the American Type Culture Collection (ATCC, Manassas, VA, USA, Catalog #TIB-1613). The cells were cultured in Dulbecco’s modified Eagle’s medium (DMEM) + glutamine + sodium pyruvate (Mediatech, Manassas, VA, USA) containing 10% FBS (Atlanta Biologicals, Atlanta, GA, USA), supplemented with 100 μg/mL streptomycin and 100 units/mL penicillin (Gibco, Gaithersberg, MD, USA) at 37 °C and 5% CO_2_. Cells were then plated at 10,000–20,000 cells per well in a 96-well plate and immediately dosed with either medium alone, ethanol, or 10 μM of either bexarotene or an analog in a total volume of 200 μL. After incubating for 72 h, 20 μL of CellTiter 96 AQueous One Solution Cell Proliferation Assay (MTS) (Promega) was added to each well, and the plate was incubated at 37 °C according to the manufacturer’s recommendations. MTS assays were quantified using a BioTek ELx808 96-well plate reader (BioTek, Winooski, VT, USA) at 490 nm. The percentage of proliferation inhibition was calculated using the value for the medium control set to 0.0 as the negative control.

### 2.4. Quantitative Real-Time PCR

Hut78 cells were maintained in DMEM/high glucose, L-glutamine, and sodium pyruvate (Cytiva Hyclone, Marlborough, MA, USA) containing 10% FBS (Atlanta Biologicals), supplemented with 100 μg/mL streptomycin and 100 units/mL penicillin (Gibco) at 37°Celsius, 5% CO_2_. Cells were plated at 400,000 cells per well in a 6-well plate. After 24 h, the medium was replaced with DMEM containing 1% FBS for 24 h. The cells were dosed in DMEM/1% FBS containing ethanol, bexarotene, or an analog for 24 h. Total RNA was isolated from each well using an Aurum Total RNA Mini Kit (Bio-Rad, Hercules, CA, USA) according to the manufacturer’s instructions. The RNA obtained was quantified using A260/280 spectrophotometry. DNase-treated total RNA (0.1 μg) was reverse-transcribed via the use of the AzuraQuant Green 1-Step qPCR Mix LoRox 1000 Reactions kit (*Azura Genomics*) to prepare 62 μL of first-strand cDNA synthesis and real-time PCR components. Reactions were prepared by adding 31 μL of 2× AzuraQuant 1-step Green LoRox, 1.55 μL of forward/reverse primers (18 μM), 3.1 μL of 20× AzuraSprint Rtase, DNase-treated total RNA (0.1 μg), and PCR-grade water for a total volume of 10 μL per well. Reactions were performed in 96-well plates in a BioRad CFX96 thermal cycler using a 40-cycle profile. Data analysis was performed using the comparative ΔΔCt method as the means of relative quantitation, normalized to an endogenous reference (GAPDH) and relative to a calibrator (normalized Ct value from vehicle-treated cells) and expressed as 2−ΔΔCt according to Applied Biosystems’ User Bulletin 2, revision B, “Relative Quantitation of Gene Expression”. The primers utilized during PCR experimentation are as follows: human GAPDH forward, 5′-ACAACTTTGGTATCGTGAAGGAC3′; human GAPDH reverse, 5′-CAGGGATGATGTTCTGGAGAGC-3′; human EGR3 forward 5′-CAATCTGTACCCCGAGGAGA-3′; human EGR3 reverse 5′-GGAAGGAGCCGGAGTAAGAG-3′; human ATF3 forward 5′-GAGGATTTTGCTAACCTGACGC-3′; human ATF3 reverse 5′-CTACCTCGGCTTTTGTGATGG-3′. Each assay was performed a minimum of three separate times for biological replicates.

### 2.5. Data Analysis

Statistical differences between the two groups (the bexarotene control treatment versus rexinoid treatment) were determined by a two-sided Student’s *t*-test, performed in Microsoft Excel. All error bars represent the standard deviation. Data points without error bars have standard deviations below Excel’s limit to display. A *p*-value of less than or equal to 0.05 was considered significant.

### 2.6. Cytotoxicity and Mutagenicity

Cytotoxicity and mutagenicity were performed as described in [35]. Ethidium bromide was used as a positive control and DMSO was used as a negative control.

### 2.7. HPLC, NMR, and High-Resolution Mass Spectrometry

All tested compounds were assessed on a Waters Acquity UPLC with QDA and PDA detectors. Compounds were assayed in ESI-mode on an ACE Excel C18-PFP (1.7 μm, 50 mm × 2.1 mm) column using a 0.1% formic acid/water:acetonitrile gradient over 5 min. A 400 MHz Bruker Avance III spectrometer was used to acquire ^1^H NMR and ^13^C NMR spectra. All synthetic procedures and experimentals, as well as NMR spectra and HPLC traces, for all compounds described in this work are described in our previous work [35] and are freely available.

### 2.8. Principal Component Analysis

PCA was performed using ClustVis [39] utilizing chemical properties of the compounds as outlined in Supplemental Table S1.

## 3. Results

### 3.1. Biological Evaluation of Generation 6 Analogs (***A64***–***A68***) via an M2H Luciferase-Based System

In order for a rexinoid to carry out its therapeutic effects as a modulator of genes involved in cell differentiation, proliferation, and apoptosis, it must first induce RXR-RXR homodimerization [26]. Therefore, a mammalian-2-hybrid (M2H) luciferase assay was employed to determine the efficacy of RXR-RXR homodimerization induced by our analogs compared to bexarotene. In this assay, HEK cells were transfected with the plasmid components of the M2H system (see Methods), and the cells were subsequently dosed with either ethanol (vehicle), 100 nM bexarotene, or the indicated analog for 24 h. Subsequently, transcription of the luciferase gene, an index that is directly proportional to the amount of RXR-RXR homodimerization, was measured via luminescence. The concentration of ligands used in these studies (100 nM) was based on preliminary data that assessed the EC_50_ of these compounds which are all in the range of 50–200 nM.

**Figure 2:**
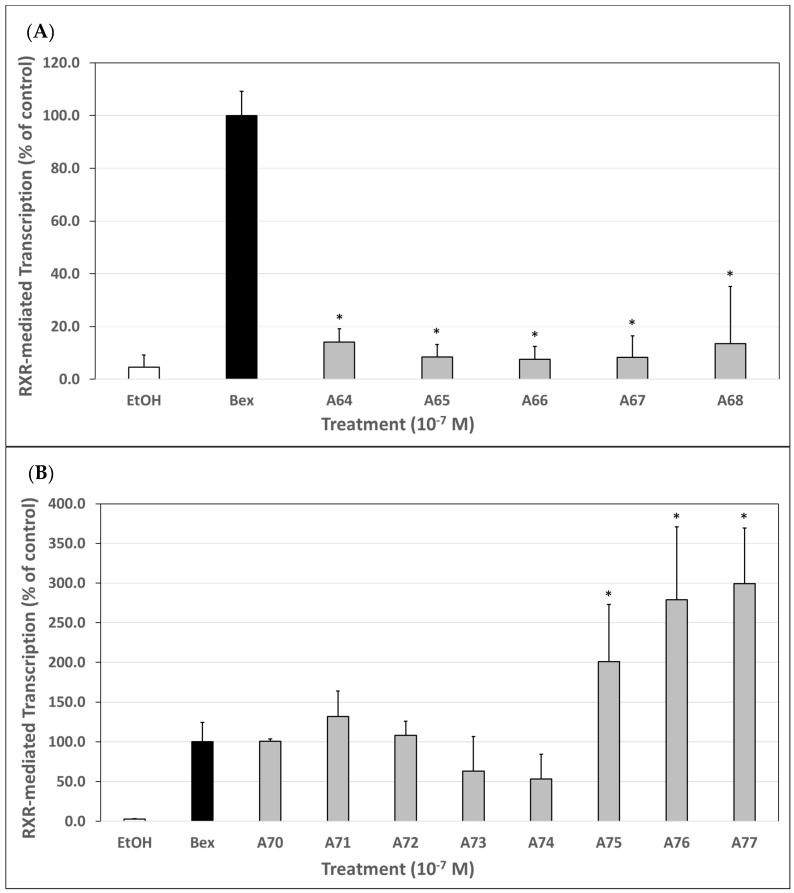
(**A**). Biological evaluation of generation 6 (**A64**–**A68**) RXR agonists via an M2H luciferase-based system. Human embryonic kidney cells (HEK293) were co-transfected using a human RXR binding domain (BD) vector (bait), a human RXR activation domain (AD) vector (prey), pFR-Luc, and renilla control plasmids for 24 h utilizing a liposome-mediated transfection protocol. Cells were treated with either the ethanol vehicle or 100 nM of bexarotene or the indicated analog for 24 h. After 24 h, the cells were lysed, and a luciferase assay was completed. Analog-dependent RXR binding and homodimerization, as measured by luciferase output, was compared to bexarotene (value set to 100%). Values are means ± SD with all analogs tested displaying lower RXR homodimerization activity vs. bexarotene (* *p* < 0.05). (**B**). Biological evaluation of generation 7 (**A70**–**A77**) RXR agonists via an M2H luciferase-based system. Human embryonic kidney cells (HEK293) were co-transfected using a human RXR-binding domain (BD) vector (bait), a human RXR-activation domain (AD) vector (prey), pFR-Luc, and renilla control plasmids for 24 h utilizing a liposome-mediated transfection protocol. Cells were treated with either the ethanol vehicle or 100 nM of bexarotene or the indicated analog for 24 h. After 24 h, the cells were lysed, and a luciferase assay was completed. Analog-dependent RXR binding and homodimerization, as measured by luciferase output, was compared to bexarotene (value set to 100%). Values are means ± SD with **A75**–**A77** displaying enhanced RXR homodimerization activity vs. bexarotene (* *p* < 0.05), whereas **A70**–**A74** displayed comparable activity vs. bexarotene.

In this first set of experiments, the homodimerization and subsequent transcriptional activity of generation 6 analogs were compared to bexarotene, which was set to 100%. The transcriptional activity of these analogs ranged from 7.5% to 14.1% of the bexarotene control. All of the generation 6 analogs, as well as the standard **A64** compound, exhibited significantly less RXR-mediated activity in the M2H luciferase-based assay (Figure 2A) than bexarotene at 100 nM.

### 3.2. Biological Evaluation of Generation 7 Analogs (***A70***–***A77***) via an M2H Luciferase-Based System

In a parallel set of experiments, we employed the same M2H luciferase assay as described above, but instead compared the generation 7 analogs to bexarotene, set to 100%. The transcriptional activity of the analogs ranged from 53.3% to 299.4% of the bexarotene control. Within generation 7 analogs (Figure 1), **A75** [37] and **A76** [38] served as standards for comparison with all other generation 7 analogs (**A70**–**A74**, and **A77**) whose synthesis and characterization we have reported [35]. Analogs **A70**–**A72**, and **A75**–**A77** all demonstrated higher RXR homodimerization in this assay (Figure 2B). Of these, **A75**–**A77** displayed statistically significant (*p* < 0.01) improvement in driving RXR-RXR homodimerization compared to bexarotene with analog **A77** exhibiting the highest activity of all compounds tested.

### 3.3. Assessment of Generation 6 Analogs (***A64***–***A68***) via an RXRE Luciferase-Based System

**Figure 3:**
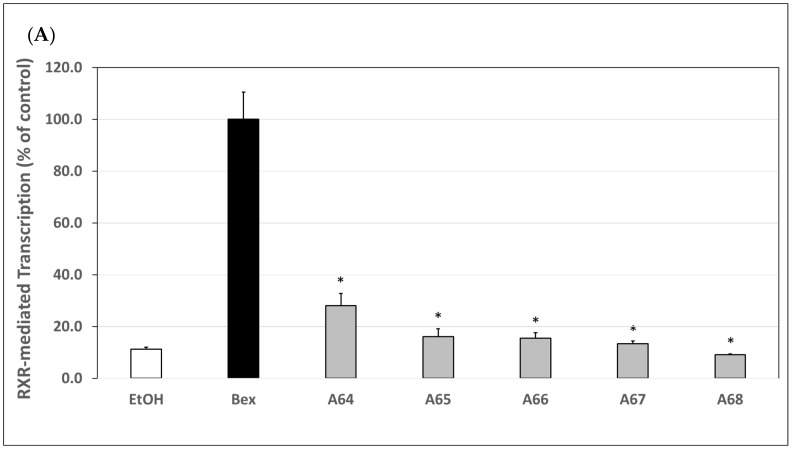

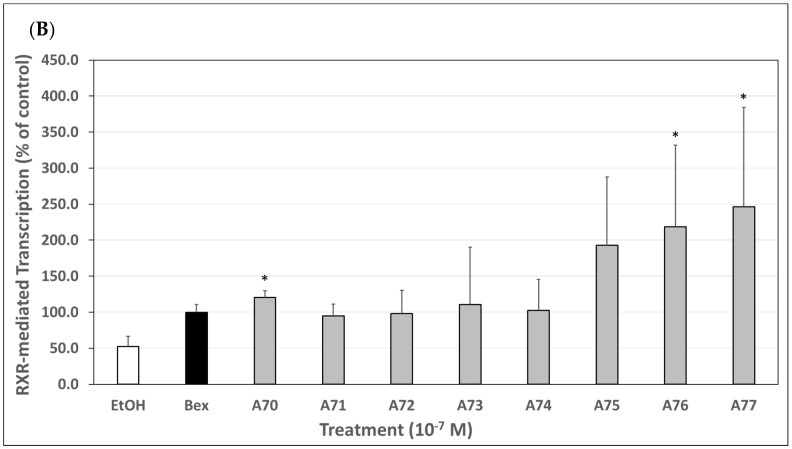
(**A**). Biological evaluation of generation 6 (**A64**–**A68**) RXR agonists via an RXRE luciferase-based system. Human embryonic kidney cells (HEK293) were co-transfected using a RXRE luciferase reporter gene, pSG5-human RXRα, and a renilla control plasmid for 24 h utilizing a liposome-mediated transfection protocol. Cells were treated with either the ethanol vehicle or 100 nM of bexarotene or the indicated analog for 24 h. After 24 h, the cells were lysed, and a luciferase assay was completed. Analog-dependent RXR-mediated transcription, as measured by luciferase output, was compared to bexarotene (value set to 100%). Values are means ± SD with **A64**–**A68** displaying lower RXR-mediated transcriptional activity vs. bexarotene (* *p* < 0.05). (**B**). Biological evaluation of generation 7 (**A70**–**A77**) RXR agonists via an RXRE luciferase-based system. Human embryonic kidney cells (HEK293) were co-transfected using a RXRE luciferase reporter gene, pSG5-human RXRα, and a renilla control plasmid for 24 h utilizing a liposome-mediated transfection protocol. Cells were treated with either the ethanol vehicle or 100 nM of bexarotene or the indicated analog for 24 h. After 24 h, the cells were lysed, and a luciferase assay was completed. Analog-dependent RXR-mediated transcription, as measured by luciferase output, was compared to bexarotene (value set to 100%). Values are means ± SD with **A70**, **A76**, and **A77** displaying enhanced RXR-mediated transcriptional activity vs. bexarotene (* *p* < 0.05), whereas **A71**–**A75** displayed comparable activity vs. bexarotene.

After RXR-RXR homodimerization takes place, the complex must then associate with the appropriate response element, the RXRE, to carry out transcriptional regulation. To assess this next molecular step in the pathway of rexinoid signaling, we utilized an RXRE luciferase assay where transcription of the luciferase gene is directly proportional to RXR-RXR homodimer binding to RXRE. HEK cells were transfected with a plasmid containing an authentic RXRE DNA sequence upstream of the luciferase gene. The transcriptional activity of generation 6 analogs was compared to bexarotene set to 100%. The activity of our analogs ranged from 9% to 28% of the bexarotene control (Figure 3A) and was statistically significantly (*p* < 0.05) lower than the bexarotene control.

Expectedly, the overall trend seen in the results of the M2H assay very closely mimics what was measured in the RXRE assay, as RXR-RXR homodimerization and homodimer binding to the RXRE are consecutive molecular functions necessary for rexinoids to produce their pharmacologic effects.

### 3.4. Assessment of Generation 7 Analogs (***A70***–***A77***) via an RXRE Luciferase-Based System

We then employed the RXRE assay, comparing generation 7 analogs to bexarotene. The transcriptional activity of these analogs ranged from 94.7% to 246.3% of the bexarotene control (Figure 3B).

Analogs **A70** and **A75**–**A77** all displayed a greater activity trend than bexarotene, and **A70**, **A76**, and **A77** revealed a statistically significant (*p* < 0.05) difference compared to the bexarotene control. Again, the trend in these results closely mimics what was observed in the M2H assay, indicating that these two assays assess complementary molecular mechanisms.

### 3.5. Biological Appraisal of Generation 6 and 7 Analogs via a CTCL Cell Proliferation Assay

**Figure 4:**
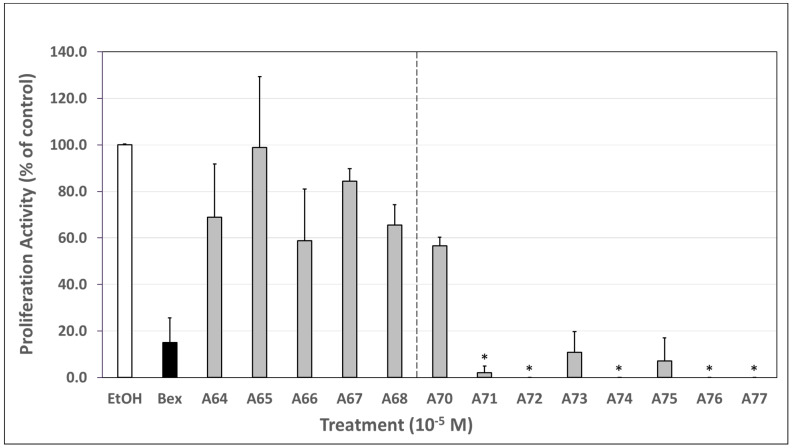
Biological evaluation of generation 6 (**A64**–**A68**) and generation 7 (**A70**–**A77**) RXR agonists via a CTCL cell proliferation assay. Human cutaneous T-cell lymphoma (Hut78) cells were plated at 10,000–20,000 cells per well and immediately dosed with either the ethanol vehicle or 10 μM of bexarotene or the indicated analog for 72 h. After 72 h, an MTS assay was performed. The RXR-mediated growth inhibition of bexarotene and each analog, measured as the inverse of the amount of light absorbed at 490 nm, was compared to that of the ethanol vehicle which was set to 100% proliferation activity to serve as the negative control. Bexarotene produced an 85% reduction in cell proliferation. Analogs **A71**, **A72**, **A74**, **A76**, and **A77** produced a complete or almost complete termination of cell proliferation that was statistically significantly lower than bexarotene (* *p* < 0.05). The data represent averages of three independent experiments.

To determine the antiproliferative efficacy of our analogs relative to bexarotene, we employed a CTCL (human Hut78) cell proliferation assay. Ethanol, the negative control, was set to 100% proliferation activity. In this set of experiments, our analogs evoked a wide range of responses in their ability to reduce the cell proliferation of Hut78 cells (Figure 4).

For example, our generation 6 analogs (**A64**–**A68**) produced a slight reduction in cell proliferation, with a mean range of 58.7–98.9% proliferation activity relative to ethanol. Treatment with bexarotene, on the other hand, led to an average of roughly 15% proliferative activity relative to ethanol. In other words, bexarotene caused an 85% reduction in cell proliferation compared to the control, much greater than all generation 6 analogs. However, most generation 7 analogs (**A70**–**A77**) displayed much larger reductions in cell proliferation with a mean range of 0–56.6% proliferation activity relative to ethanol. Of note, **A71**, **A72**, **A74**, **A76**, and **A77** produced an almost complete termination of Hut78 cell proliferation, statistically significantly lower than that achieved with bexarotene (Figure 4, *p* < 0.05). The disparity in performance of the two generations of analogs is corroborated by the M2H and RXRE luciferase assays, which also revealed a significant difference between generation 6 (**A64**–**A68**) and generation 7 (**A70**–**A77**) analogs with the exception of **A70**, which displayed a similar RXR activity to bexarotene in the M2H and RXRE luciferase assays but did not reduce Hut78 cell proliferation to a similar extent as bexarotene or other generation 7 analogs.

### 3.6. Cytotoxicity and Mutagenicity Assessment of Rexinoids

To determine the potential suitability of druggability of the rexinoids, cytotoxicity and mutagenicity were assessed [35]. The results are delineated as in Table 1. None of the compounds are mutagenic. However, **A64**–**A68** and **A77** are cytotoxic at the concentrations shown below which represent concentrations that resulted in 50% cell death. Of note, the concentrations used for testing are much higher than those used in the cellular assays, thus the cytotoxicity would not be evident in the HEK and CTCL assays.

### 3.7. qPCR Analysis of ATF3 Gene Induction

**Figure 5:**
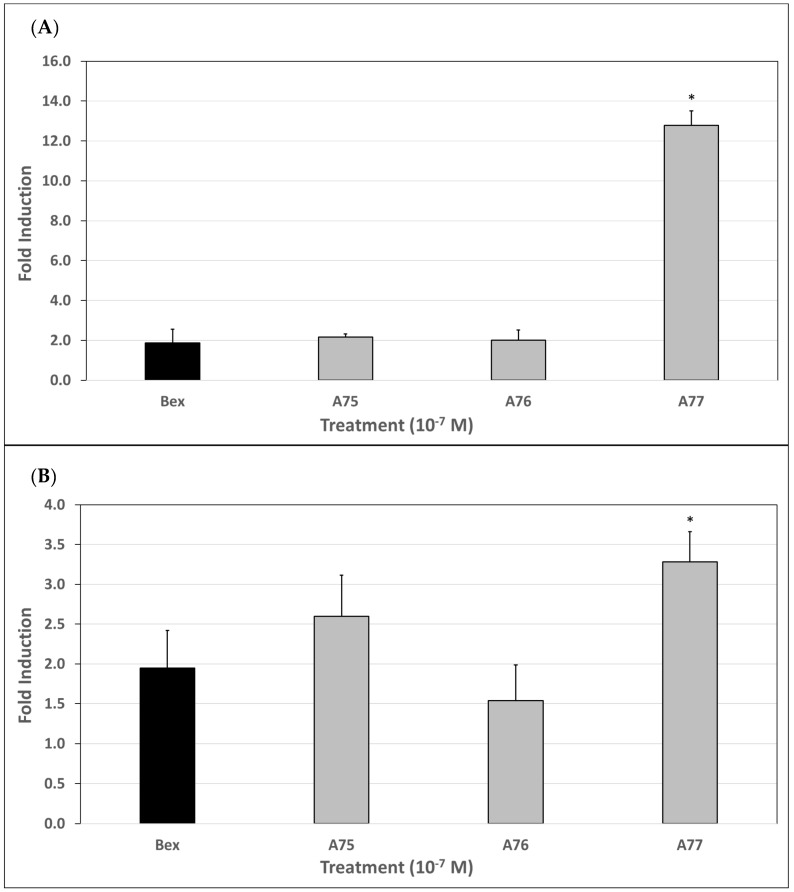
(**A**). qPCR analysis of our most potent RXR agonists (**A75**–**A77**) to induce ATF3 gene expression. Human cutaneous T-cell lymphoma (Hut78) cells were treated for 48 h with bexarotene or the indicated analog at 100 nM. A 0.1 μg portion of DNase-treated RNA was used for cDNA synthesis and subsequent qPCR. Data analysis was performed using the comparative ΔΔCt method as the means of relative quantitation, normalized to an endogenous reference (GAPDH) and relative to a calibrator (normalized Ct value from vehicle-treated cells) and expressed as 2^−ΔΔCt^. * indicates *p* < 0.001 vs. bexarotene. Results are from at least three independent biological replicates. (**B**). qPCR analysis of our most potent RXR agonists (**A75**–**A77**) to induce EGR3 gene expression. Human cutaneous T-cell lymphoma (Hut78) cells were treated for 48 h with bexarotene or the indicated analog at 100 nM. A 0.1 μg portion of DNase-treated RNA was used for cDNA synthesis and subsequent qPCR. Data analysis was performed using the comparative ΔΔCt method as the means of relative quantitation, normalized to an endogenous reference (GAPDH) and relative to a calibrator (normalized Ct value from vehicle-treated cells) and expressed as 2^−ΔΔCt^. * indicates *p* < 0.05 vs. bexarotene. Results are from at least three independent biological replicates.

qPCR analysis was performed to determine efficacy of these analogs to upregulate gene transcription of the tumor suppressor gene, ATF3, relative to bexarotene. Since not all analogs displayed robust activity in the previous biological assays, only the most potent analogs, namely **A75**–**A77**, were selected for qPCR evaluation (Figure 5A).

In this set of experiments, bexarotene exhibited a mean 1.9-fold increase in ATF3 gene transcription relative to the ethanol control. **A75** and **A76** yielded remarkably similar and not statistically significant fold inductions to bexarotene at 2.2 and 2.0, respectively. **A77**, on the other hand, induced a mean fold increase of 12.8 over that of the ethanol control. Therefore, based on these results, **A77** is 6.9 times more effective than bexarotene in the induction of ATF3 (Figure 5A, *p* < 0.001).

### 3.8. qPCR Analysis of EGR3 Gene Induction

qPCR analysis was performed to determine efficacy of our analogs to upregulate gene transcription of another known tumor suppressor gene, EGR3, relative to bexarotene. Again, only the analogs with the highest activity, namely **A75**–**A77**, were selected for qPCR evaluation (Figure 5B).

In this set of experiments, bexarotene showed a mean 2.0-fold increase in EGR3 gene transcription relative to the ethanol control. Analog **A75** displayed a higher mean fold induction than bexarotene at 2.6; however, this result did not reach full statistical significance. Additionally, **A76** produced a lower mean fold induction than bexarotene at 1.5, but this result was not statistically significant. In contrast, **A77** induced EGR3 expression by 3.3-fold compared to the ethanol control, which was statistically significantly higher than bexarotene (*p* < 0.05). Therefore, **A77** is 1.7 times more effective than bexarotene in the induction of EGR3.

## 4. Discussion

In this study, we describe the evaluation of ten novel analogs and three bexarotene standards from the literature using various biological assays to assess RXR specificity, inhibition of CTCL cell proliferation, and general cytotoxicity. The most potent compounds were also analyzed via qPCR to determine efficacy in the induction of two tumor suppressor genes, ATF3 and EGR3. Our analogs were synthesized as two distinct generations, generation 6 and generation 7, maintaining specific molecular motifs within their chemical structures (Figure 1) and tested alongside relevant standards as explained in the biological evaluation of generation 6 and 7. Based on the results obtained in our experiments, most of the generation 6 analogs demonstrated less selectivity and potency as compared to bexarotene. In the M2H (Figure 2A) and RXRE (Figure 3A) assays, analogs **A64**–**A68** yielded considerably less RXR-mediated transcription of the luciferase gene than bexarotene, indicating that these analogs are less effective at inducing a response through the RXR-RXRE nuclear signaling pathway. Rexinoids produce their chemotherapeutic effects, at least in part, via this pathway which permits the modulation of genes involved in cell proliferation, differentiation, and apoptosis. Therefore, analogs **A64**–**A68** are less likely to lead to an improved therapeutic outcome in the treatment of CTCL than bexarotene. This conclusion is further substantiated by the results obtained in the proliferation assay as these analogs reduced CTCL cell proliferation to a lesser degree than did the bexarotene parent compound (Figure 4), although it is important to point out that we conducted the CTCL proliferation assays using only one established CTCL cell line (Hut78). In future studies, we will also evaluate additional CTCL cell lines such as HH and H9.

Conversely, generation 7 analogs performed far more favorably in general. In this group, all analogs yielded either comparable or improved results compared to bexarotene across the M2H, RXRE, and proliferation assays. Most notably, analogs **A75**–**A77** demonstrated an enhanced ability to induce RXR homodimerization (Figure 2B), activate RXRE (Figure 3B), and reduce CTCL cell proliferation (Figure 4) relative to bexarotene. The results suggest that these compounds, if tested with in vivo CTCL animal models [40,41,42,43,44], may produce more satisfactory outcomes in CTCL disease remission. However, **A77** was cytotoxic at the lowest concentration evaluated, so additional testing is warranted before moving onto a mouse model. Interestingly **A70**, also a 7th generation analog, performed similarly to bexarotene at 100 nM in the M2H and RXRE assays but only resulted in ~43% reduction in proliferation in the proliferation assay. However, when observing the general trend within the data, it is clear that the M2H and RXRE assays serve as powerful predictive screening tests given that the compounds which most induced RXR homodimerization and subsequent RXRE-mediated activation generally reduced CTCL cell proliferation to a proportionately greater degree.

**Figure 6:**
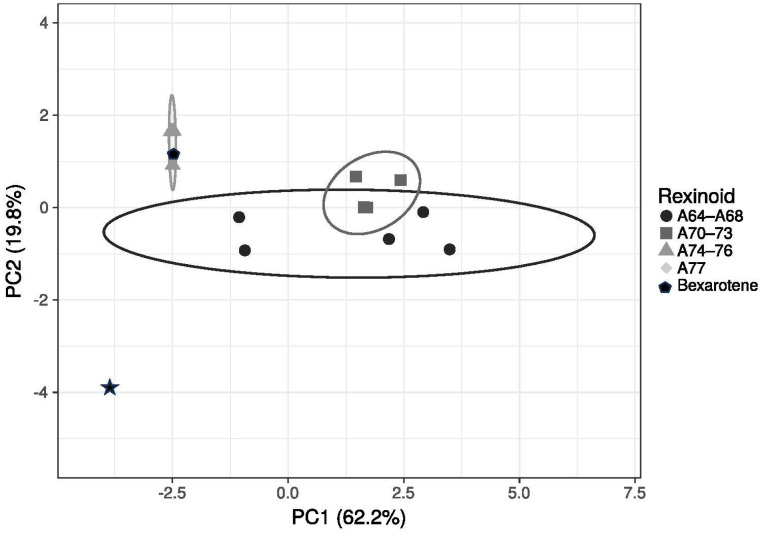
Principal component analysis of rexinoids used in this study. Chemical properties as outlined in Supplemental Table S1 were used to produce a PCA plot of the rexinoids used in this study. Unit variance scaling is applied to rows; SVD with imputation is used to calculate principal components. The X and Y axis show principal component 1 and principal component 2 that explain 62.2% and 19.8% of the total variance, respectively. Prediction ellipses are such that with a probability of 0.95, a new observation from the same group will fall inside the ellipse. N = 14 data points.

Therefore, we posit that the potential therapeutic utility of a rexinoid may be predicted based on its performance in these assays and this approach may serve as a pragmatic tool in the assessment of novel rexinoids intended for the treatment of CTCL. With consideration given to this hypothesis, we further evaluated our top performing analogs, **A75**–**A77**, via qPCR to assess for upregulation of the ATF3 and EGR3 genes. These genes were specifically selected for analysis as they are involved in tumor suppression pathways and have been previously found to be upregulated in response to retinoids and rexinoids, such as bexarotene [45,46,47]. While upregulation of these genes may not be the only mechanism by which these compounds exert their chemotherapeutic effects, we hypothesized that our most potent analogs would generate similar or enhanced fold-inductions when compared to bexarotene. In fact, **A75** and **A76** produced equipollent responses while **A77** produced statistically greater fold-inductions in both genes, especially ATF3 (Figure 5A,B). Although these results are at the level of mRNA expression, it will be important to confirm and extend these observations by performing a Western analysis to probe the level of protein expression. Nonetheless, these mRNA expression findings suggest that although bexarotene may be effective for the treatment of refractory CTCL, introducing new structural motifs (Figure 1) to the bexarotene parent compound may in fact yield analogs that are more efficacious at induction of important bio-response/target genes, and maintain an improved side effect profile due to enhanced selectivity. While several of the analogs based on the known compound **A64** [36] were also largely inactive, the known **A75** [37] and **A76** [38] compounds showed enhanced activity, and the rexinoid **A77**—an analog of bexarotene that our group designed in which a single hydrogen atom adjacent to the carboxylic acid was replaced with a hydroxyl group—shows a statistically robust enhanced activity compared to bexarotene. Our group has recently published a work in which a principal component analysis showed that certain structural motifs tended to group according to similar biological activities, and we continue to explore the potential for structure–activity correlation [48]. Indeed, enhanced PCA analysis with additional physical and chemical properties of these compounds (Figure 6) demonstrates that **A77** has unique characteristics that set it apart from the other rexinoids in this study. Analogs **A74**–**A77** all possess the lowest LogS values (similar to the LogS of bexarotene), and the lowest of the hydrogen bond acceptors of the novel rexinoids (again similar to bexarotene). Moreover, **A74**–**A77** also have the highest cLogP values, even greater than bexarotene. **A77** includes two hydrogen bond donors, compared to all the other compounds that only have one; and **A77** cLogP and cLogD values are the highest of the group. Taken together, **A77** has the most hydrogen bond donors and fewer hydrogen bond acceptors, as well as being less soluble than the other molecules. This combination of characteristics sets this rexinoid apart in both the PCA analysis as well as the biological assays, with a potent combination of elevated RXRE and M2H activity, compelling proliferation inhibition, and high-gene-expression induction of both tumor suppressor genes tested.

RXR agonists and, more specifically, bexarotene analog drug development represent a potentially valuable area of exploration. Our research group, and some others, have previously published research describing the development and biological evaluation of other rexinoids with an emphasis on treating human diseases, such as CTCL and Alzheimer’s. In 2009, our group delineated eleven novel analogs of bexarotene, three of which demonstrated similar RXR-mediated transcriptional activity and stimulation of apoptosis in a CTCL system [49]. Molecular modeling studies were also performed which identified structural motifs that possess improved binding affinities to RXR and paved the way for future analog development. More recently, our group has reported seven novel analogs of bexarotene belonging to generations 6 and 7 as described here and performed similar biological assays as presented in this study, where the results showed that all seven analogs possessed significantly less cross-over activity onto RAR-RARE signaling pathways than bexarotene, indicating improved receptor specificity and therefore a potentially reduced side effect profile if utilized as a therapy in humans [35]. Furthermore, our group has performed gene expression analytics on twelve analogs, eight of which were synthesized in our lab, in a previous study [49], investigating the differential expression of 102 genes involved in oncogenic cellular activity. We demonstrated that CTCL cells may respond differently based on exposure to distinctive rexinoids with the implication that minor variations in chemical structure may lead to unique gene expression profiles [50]. Of the analogs tested in that study, five showed promise for further analysis and drug development due to favorable gene expression signatures relative to bexarotene.

Other groups have also investigated rexinoid drug design with the purpose of finding safer and more effective alternatives to bexarotene. A novel RXR ligand, 9-cis UAB30 (UAB30) was compared to bexarotene, and the activity of UAB30 was comparable to bexarotene at inducing cell apoptosis and suppressing cell proliferation. However, it maintained a minimal effect in elevating serum triglycerides and did not induce hypothyroidism, thus making it a better alternative to bexarotene in treating CTCL [51]. Due to its role as the favored heterodimer partner for approximately one third of all human nuclear receptors, other researchers have recognized RXR and its agonists as valuable targets for research and development of chemotherapeutics. de Almeida and Conda-Sheridan evaluated several RXR agonists, including bexarotene, and discussed various templates that have been reported to activate RXR with emphasis on molecular structure and biological activity, expressing optimism for rexinoids that can be developed to minimize untoward effects [52].

## 5. Conclusions

The current study expands on previous work to develop a more specific rexinoid and develop a better understanding of the effects of more potent rexinoids on Hut78 cell proliferation. Additionally, we continue to demonstrate that changes to the molecular structure of bexarotene may lead to the development of novel rexinoids that possess enhanced efficacy and dampened side effect profiles. Currently in the United States, there are only two FDA-approved RXR agonists; alitretinoin- used as a topical treatment of Kaposi’s sarcoma, chronic hand eczema, and psoriasis, and bexarotene. Due to their ability to influence the expression of a vast number of genes involved in cellular regulatory pathways, the application of RXR agonists in the treatment of other pathologies as either a primary or adjunctive therapy should be investigated. Equally important is the improvement of the existing repertoire of RXR agonists in the treatment of diseases where these drugs are known to already be effective, as is the case with bexarotene and CTCL. Although bexarotene has been used to treat refractory CTCL with considerable success, it remains only one of a limited number of options available for patients who have failed to respond to at least one prior therapy. However, the side effects of bexarotene therapy are well documented and unfortunately quite common. As a consequence, bexarotene is concomitantly prescribed with cholesterol lowering medications such as a statin and tetraiodothyronine as a thyroid hormone replacement to ward off the sequelae of hyperlipidemia and hypothyroidism, respectively. Patients must often discontinue the use of bexarotene in order to allow lab values to normalize as hyperlipidemia and hypertriglyceridemia are risk factors for pancreatitis and cardiovascular disease [53]. These factors complicate the treatment regimen leading to increased cost, reduced compliance, and vulnerability to additional side effects from ancillary medications. It is for these reasons that we believe the development of novel bexarotene analogs to be paramount and an area deserving of further investigation. The expansion of novel rexinoids continues to be an area of focus for our laboratories, as we are currently synthesizing additional bexarotene analogs which are being analyzed in several biological assays, including those reported herein. Future studies will include these next generations of analogs with unique chemical structural motifs that include the insertion of an oxygen atom into the non-polar ring system of potent rexinoids such as bexarotene, as well as engineering the non-polar ring system to be more rigid.

With the development of more potent and selective analogs, these drugs may be prescribed at lower therapeutic doses which would theoretically lead to a decrease in side effects. Furthermore, possessing a broader array of FDA-approved rexinoids will allow clinicians to tailor treatment regimens based on a patient’s risk factors, genetics, and the medication’s side effect profile.

## Figures and Tables

**Table 1:** Assessment of cytotoxicity and mutagenicity of bexarotene, generation 6 (**A64**–**A68**) and generation 7 (**A70**–**A77**). The indicated compounds are cytotoxic and demonstrate 50% cell death on Petri dishes at the concentrations shown; none of the analogs are mutagenic.

Compound	Cytotoxicity
Bexarotene	None
**A64**	0.5 mg/mL
**A65**	0.5 mg/mL
**A66**	1 mg/mL
**A67**	1 mg/mL
**A68**	0.08 mg/mL
**A70**	None
**A71**	None
**A72**	None
**A73**	None
**A74**	None
**A75**	None
**A76**	None
**A77**	0.08 mg/mL

## Data Availability

Data is contained within the article or Supplementary Material.

## Supplementary Materials

The following supporting information can be downloaded at: https://www.mdpi.com/article/10.3390/cells12212575/s1, Table S1: Chemical Properties Used in PCA Analysis (Figure 6). Calculation of HBA, HBD, TPSA, LogS, and cLogP was performed using DataWarrior [54]. Calculation of LogP and water solubility were performed using SwissADME [55]. cLogD was calculated using Chemaxon Software (Chemaxon, San Diego, CA). The CNS MPO Score (denoted as Score) was calculated using the algorithm in [56]. Violations of Lipinski’s Rules [57] were calculated using existing data from the table.
