# Supplementary material for: Development of Bexarotene Analogs for Treating Cutaneous T-Cell Lymphomas
Source: Cells. 2023 Nov 4;12(21):2575. doi: 10.3390/cells12212575 (PMC10647404; doi:10.3390/cells12212575)
Supplement: Supplementary file 1 [file cells-12-02575-s001.zip › cells-2672074-supplementary.pdf]

Supplemental Table S1

	**Bex**	**A64**	**A65**	**A66**	**A67**	**A68**	**A70**	**A71**	**A72**	**A73**	**A74**	**A75**	**A76**	**A77**
MW	348.48	356.47	357.45	355.48	373.21	353.17	350.2	336.18	338.45	350.46	352.18	334.45	334.46	364.2
HBD	1	1	1	1	1	1	1	1	1	1	1	1	1	2
HBA	2	5	6	4	4	5	4	4	4	4	2	2	2	3
cLogP	5.86	4.209	4.556	5.983	6	5.072	5.101	5.177	4.83	5.021	6.57	6.427	6.496	7.287
Violations	1	0	0	1	1	1	1	1	0	1	1	1	1	1
cLogD	3.835	1.86	1.21	3.28	2.91	1.73	1.79	1.84	1.59	1.71	3.15	3.31	3.38	3.79
TPSA	37.3	62.66	75.55	49.77	49.77	77.24	63.08	63.08	63.08	63.08	37.3	37.3	37.3	57.53
LogS	-7.62	-5.443	-5.467	-6.062	-6.315	-5.207	-4.896	-5.254	-5.122	-4.896	-7.603	-7.345	-7.345	-7.397
Water Sol	1	2	2	1	1	2	2	2	2	2	1	1	1	1
Score	3.8	4.4	4.2	3.6	3.7	4	4	4	4.1	4	3.8	3.7	3.7	2.3

Supplemental Table S1. Chemical Properties Used in PCA Analysis (Figure 6). Calculation of HBA, HBD, TPSA, LogS, and cLogP was performed using DataWarrior (Sander, T). Calculation of LogP and water solubility were performed using SwissADME (2). cLogD was calculated using Chemaxon Software (Chemaxon, San Diego, CA). The CNS MPO Score (denoted as Score) was calculated using the algorithm in (3). Violations of Lipinski's Rules (4) were calculated using existing data from the table.

Supplement References.

1. Sander T., Freyss, J., von Korff, M., Rufener, C. 2015. DataWarrior: An Open-Source Program For Chemistry Aware Data Visualization And Analysis. J. Chem. Info. Model. 55 (2), 460-473. DOI: 10.1021/ci500588j
2. Daina, A., O. Michielin, O., Zoete, V. 2017. SwissADME: a free web tool to evaluate pharmacokinetics, drug-likeness and medicinal chemistry friendliness of small molecules. Sci. Rep. 7:42717. doi: 10.1038/srep42717.
3. Wager, T.T., Hou, X., Verhoest, P.R., Villalobos, A. 2016. Central Nervous System Multiparameter Optimization Desirability: Application in Drug Discovery. ACS Chem. Neuro. 7 (6), 767-775. DOI: 10.1021/acschemneuro.6b00029
4. Lipinski CA. Drug-like properties and the causes of poor solubility and poor permeability. J. Pharmacology. Toxicol. Methods. 2000;44:235–249.
